# Differential Modulation of Effective Connectivity in the Brain’s Extended Face Processing System by Fearful and Sad Facial Expressions

**DOI:** 10.1523/ENEURO.0380-20.2021

**Published:** 2021-04-08

**Authors:** Alec J. Jamieson, Christopher G. Davey, Ben J. Harrison

**Affiliations:** 1 Melbourne Neuropsychiatry Centre, Department of Psychiatry, The University of Melbourne and Melbourne Health, Carlton, Victoria 3053, Australia; 2 Department of Psychiatry, The University of Melbourne, Parkville, Victoria 3052, Australia

**Keywords:** dynamic causal modeling, effective connectivity, emotion processing, fMRI, youth

## Abstract

The processing of emotional facial expressions is underpinned by the integration of information from a distributed network of brain regions. Despite investigations into how different emotional expressions alter the functional relationships within this network, there remains limited research examining which regions drive these interactions. This study investigated effective connectivity during the processing of sad and fearful facial expressions to better understand how these stimuli differentially modulate emotional face processing circuitry. Ninety-eight healthy human adolescents and young adults, aged between 15 and 25 years, underwent an implicit emotional face processing fMRI task. Using dynamic causal modeling (DCM), we examined five brain regions implicated in face processing. These were restricted to the right hemisphere and included the occipital and fusiform face areas, amygdala, and dorsolateral prefrontal cortex (dlPFC) and ventromedial prefrontal cortex (vmPFC). Processing sad and fearful facial expressions were associated with greater positive connectivity from the amygdala to dlPFC. Only the processing of fearful facial expressions was associated with greater negative connectivity from the vmPFC to amygdala. Compared with processing sad faces, processing fearful faces was associated with significantly greater connectivity from the amygdala to dlPFC. No difference was found between the processing of these expressions and the connectivity from the vmPFC to amygdala. Overall, our findings indicate that connectivity from the amygdala and dlPFC appears to be responding to dimensional features which differ between these expressions, likely those relating to arousal. Further research is necessary to examine whether this relationship is also observable for positively valenced emotions.

## Significance Statement

While previous research has implicated interactions between the amygdala and prefrontal regions as important to the processing of emotional stimuli, limited investigations into the directional interactions of these regions exist. Our findings highlight differences between the implicit processing of sad and fearful facial expressions in the connectivity from the amygdala to dorsolateral prefrontal cortex (dlPFC). By refining our models of the brain network dynamics in healthy individuals, this work may enable us to better understand how this network becomes dysfunctional in neurological and mental health disorders marked by altered emotion processing.

## Introduction

The ability to comprehend the emotions of others through facial expressions is central to human social interactions (Frith, 2009). This process is supported by a distributed network of brain regions, the so-called “face processing network,” which has been extensively detailed through neuroimaging research (Fairhall and Ishai, 2007; Palermo and Rhodes, 2007; Haist and Anzures, 2017). Previous research has typically divided the face processing network into “core” and “extended” systems (Haxby et al., 2000; Gschwind et al., 2012). The core system, including the occipital and fusiform face areas (OFA and FFA), is believed to be involved in processing facial components and incorporating these parts into a holistic representation (Liu et al., 2010; Jiedong et al., 2012). In contrast, regions of the extended system including the amygdala, dorsolateral prefrontal cortex (dlPFC) and ventromedial prefrontal cortex (vmPFC) appear to be important in integrating this basic information with higher-order functions (Adolphs, 2002, 2008; Ishai, 2008). As such, the extended system overlaps with many large-scale cortical networks which contribute to a range of cognitive and emotional processes (Sridharan et al., 2008).

The amygdala is a region traditionally associated with the processing of fearful stimuli; however, meta-analyses have demonstrated increased amygdala activity for both positive and negative valenced facial expressions (Fusar-Poli et al., 2009). Studies have hypothesized that the amygdala’s role in face processing is to respond to novel and highly salient information (Blackford et al., 2010; Todorov, 2012; Jacob et al., 2014). This is consistent with the finding of greater activity during the processing of fearful compared with sad expressions (Fusar-Poli et al., 2009), as although these expressions are both negatively valenced, they differ in ratings of arousal (Hedger et al., 2015; Lin et al., 2016). Moreover, increased connectivity between the amygdala and dlPFC has been commonly identified in face processing tasks (Dannlowski et al., 2009; Comte et al., 2016; Haller et al., 2018). Given their roles, the interaction between the dlPFC and amygdala, depending on the directionality, may be important in directing conscious awareness toward and regulating emotional responses to salient emotional stimuli (Dolcos et al., 2006; Banks et al., 2007; Costafreda et al., 2008; Etkin et al., 2015). Despite this, previous research examining the directionality of these interactions has not examined whether facial expressions with differing arousal ratings, such as fearful and sad expressions, differently modulate this relationship (Sladky et al., 2015; Vai et al., 2015; Willinger et al., 2019). The sparse anatomic connectivity between these regions additionally suggests that the regulatory role of the dlPFC on the amygdala is likely dependent on interactions with mediatory regions including the vmPFC (Phillips et al., 2003; Ray and Zald, 2012).

The vmPFC has been consistently implicated in the processing of emotional expressions (Heberlein et al., 2008; Hiser and Koenigs, 2018). Previous studies have reported both vmPFC activation and deactivation (Yang et al., 2019); however, the decreased activity observed during implicit emotional processing tasks is consistent with its involvement in the default mode network (Raichle et al., 2001; Harrison et al., 2008, 2011; Uddin et al., 2009). Negatively valenced expressions, particularly sad expressions, have demonstrated greater vmPFC deactivation in comparison to happy expressions (Sreenivas et al., 2012). While this implies that the vmPFC is sensitive to emotional valence, other research suggests that the vmPFC may also be sensitive to emotional arousal (Zhang et al., 2014; Kuniecki et al., 2018). As the interaction between the vmPFC and amygdala has been implicated as crucial in regulating appropriate behavioral responses to emotional stimuli, changes to the valence and arousal of these stimuli likely influence this regulation (Hartley and Phelps, 2010; Milad and Quirk, 2012; Motzkin et al., 2015; Braunstein et al., 2017). A recent study by Willinger et al. (2019) examined the effects of negatively and positively valenced facial expressions on the directional interactions between these regions. Although they highlighted that negatively valenced expressions were associated with greater negative modulation from the vmPFC to amygdala, expressions within the negatively valenced category were not compared. As a result of this paucity, whether expressions which differ in their arousal differentially alter the connectivity of the extended face processing system lacks clarification.

The present study aimed to investigate the nature of functional interactions between key components of the face processing network during the processing of negatively valenced expressions. We chose to focus on fearful and sad facial expressions because of their differences in arousal ratings (Langner et al., 2010) and the importance of negative affective processing to models of psychopathology (Palazidou, 2012; Hiser and Koenigs, 2018). We assessed functional interactions using dynamic causal modeling (DCM), an established method of assessing the effective connectivity of the brain (Friston et al., 2003). Effective connectivity is defined as the directional influence of a neural system or brain region over another (Friston, 2011). We recruited a large sample of adolescents and young adults, as this developmental period is particularly sensitive to the processing of negative emotional stimuli, including emotional faces (Vetter et al., 2015; Yuan et al., 2015).

We expected that emotional face matching would result in significant activation of the inferior occipital gyrus, fusiform gyrus, amygdala, and middle frontal gyrus, as well as deactivation of the vmPFC (Harrison et al., 2011). We hypothesized that there would be significant (1) positive modulation of the connectivity from the amygdala to dlPFC, and (2) negative modulation of the connectivity from the vmPFC to amygdala, during the processing of both sad and fearful facial expressions. We also hypothesized that (3) fearful face processing would lead to more pronounced effects on the interactions in the extended system given the greater salience and arousal of these stimuli, although we had no clear hypothesis as to the directionality of these effects.

## Materials and Methods

### Participants

Ninety-eight participants completed the study protocol after responding to online advertisements. They were between 15 and 25 years of age, had no past diagnoses of mental illness in accordance with the Structured Clinical Interview for DSM-IV Axis I Disorders (First et al., 1997, 2002) criteria, and had an IQ of >85 as assessed by the Wechsler Test of Adult Reading (Wechsler, 2001). Each participant signed an informed consent form to participate in the study (this was also done by parents of participants under the age of 18), which had been approved by the Melbourne Health Human Research and Ethics Committee. Of the original sample, six participants were omitted. These were excluded because of incidental findings (two participants), poor task performance (lower than an average 80% accuracy across all conditions; two participants), or excessive head motion (see further; two participants). Thus, 92 participants (56.5% female) with a mean age of 20.1 years (SD 2.9 years) were included in our analyses.

### Experimental design

#### Implicit emotional face matching task

The fMRI task was a variation of the face matching task first described by Hariri et al. (2000). It involved three conditions: one shape matching and two implicit face processing conditions, involving either fearful or sad facial expressions. In the shape matching condition, participants were required to match the orientation of the shape presented in the top half of the screen to one of the two shapes presented on the left and right in the bottom half of the screen. Similarly, in the two face processing conditions, participants were required to match the gender of the target face, presented in the top half of the screen, with the gender of one of the faces presented on the left and right in the bottom half of the screen. All three faces within a trial (one in the top half and two in the bottom half) displayed the same facial expression. Each block would convey either a sad or fearful facial expression. Gender matching was chosen as the main component of the task rather than emotion matching as it more closely replicates natural processes; people typically process expressions incidentally rather than being required to specifically identify them.

The order in which the conditions were presented was counterbalanced between participants [either version A (shapes, sad, fearful) or B (shapes, fearful, sad)]. Each session involved six blocks for each of the three conditions (18 blocks total), a 10-s white fixation cross was also presented between each block, and before the first and after the final block. Each block consisted of six trials, with each trial having a duration of 3.75 s followed by a 0.25-s intertrial interval. For the face processing blocks, these trials comprised three male and three female faces, which were sampled from a total of 18 male and 18 female faces.

All of the face stimuli were collected from the Radboud Face Database (Langner et al., 2010). The task was presented with Paradigm software (http://www.paradigmexperiments.com) and ran on a Dell computer. The LCD screen that presented stimuli was visible via a reverse mirror mounted to the participants’ head coil and behavioral responses were captured using an optical-fiber button-box. Differences in reaction time (RT) and accuracy between conditions were compared through the use of a repeated measures ANOVA and Friedman test, respectively, with a Holms-Bonferroni correction to adjust for multiple comparisons (Holm, 1979).

#### Image acquisition

A 3T General Electric Signa Excite system with an eight-channel phased-array head coil was used in combination with ASSET parallel imaging. The functional sequence consisted of a single shot gradient-recalled echoplanar imaging sequence in the steady state (repetition time, 2000 ms; echo time, 35 ms; and pulse angle, 90°) in a 23-cm field-of-view, with a 64 × 64-pixel matrix and a slice thickness of 3.5 mm (no gap). Thirty-six interleaved slices were acquired parallel to the anterior-posterior commissure line with a 20° anterior tilt to better cover ventral prefrontal brain regions. The total sequence duration was 10 min and 32 s, corresponding to 311 whole-brain echoplanar imaging volumes. The first four volumes from each run were automatically discarded to allow for signal equilibration. A T1-weighted high-resolution anatomic image was acquired for each participant to assist with functional time-series coregistration (140 contiguous slices; repetition time, 7.9 s; echo time, 3 s; flip angle, 13°; in a 25.6-cm field-of-view, with a 256 × 256-pixel matrix and a slice thickness of 1 mm). To assist with noise reduction and head immobility, all participants used earplugs and had their heads supported with foam-padding inserts.

### Image analysis

#### Preprocessing

Imaging data were transferred to a Unix-based platform that ran MATLAB version 9.3 (The MathWorks Inc.) and Statistical Parametric Mapping (SPM) version 12-v7487 (Wellcome Trust Centre for Neuroimaging, London, United Kingdom). Motion correction was performed by aligning each participant’s time series to the first image using least-squares minimization and a six-parameter rigid-body spatial transformation. Motion fingerprint (SPM toolbox; Wilke, 2012) was used to quantify participant head motion. Participants were excluded if movement exceeded 3 mm mean total displacement or maximum scan-to-scan displacement (approximately one native voxel; Johnstone et al., 2006; Nemani et al., 2009). Following this, images were corrected for differences in slice acquisition time and then coregistered to their respective T1 weighted scans, which had been spatially normalized and segmented using the International Consortium for Brain Mapping template. These functional images were resliced to 2-mm isotropic resolution and were smoothed with a 5-mm Gaussian kernel (full width at half maximum).

#### General linear modeling (GLM)

Each participant’s preprocessed time series was included in a first-level GLM analysis in SPM12. This was done by specifying the durations and onsets of each shape, sad, and fearful face matching blocks, respectively, to be convolved with a canonical hemodynamic response function. Each condition was modeled separately, with rest-fixation blocks forming the implicit baseline. A high-pass filter (1/128 s) accounted for low-frequency noise, while temporal autocorrelations were estimated using a first-order autoregressive model. Primary contrast images were estimated to examine responses to fearful (fearful faces > shapes) and sad faces (sad faces > shapes), as well as overall responses to these faces (sad and fearful faces > shapes), and were carried forward to the group-level using the summary statistics approach to random-effects analyses. At the group-level, single sample *t* tests were conducted, which were thresholded with a whole-brain, family-wise error rate (FWE) corrected threshold of *p *<* *0.05, K_E_ ≥ 30 voxels.

### DCM

#### Overview

DCM uses a set of differential equations and generative models to estimate interactions between neural populations from neuroimaging data (Friston et al., 2003; Friston and Penny, 2011). In contrast to functional connectivity measures which assess the statistical dependencies between different regions, this effective connectivity models the influence that one region exerts over another (for a detailed comparison of these methods, see Friston, 2011). DCM shows both how these connections behave intrinsically (invariant connectivity in the absence of task modulation) and because of the modulation induced by experimental stimuli. This is conducted by specifying and estimating the parameters for hypothetical models of neural interactions, then comparing the relative evidence of these models through Bayesian model comparison (Zeidman et al., 2019). These connectivity parameters can be either positive or negative, thus revealing that an increase in one region results in an increase or decrease, respectively, in another region.

#### Time-series extraction

**Figure 1.**
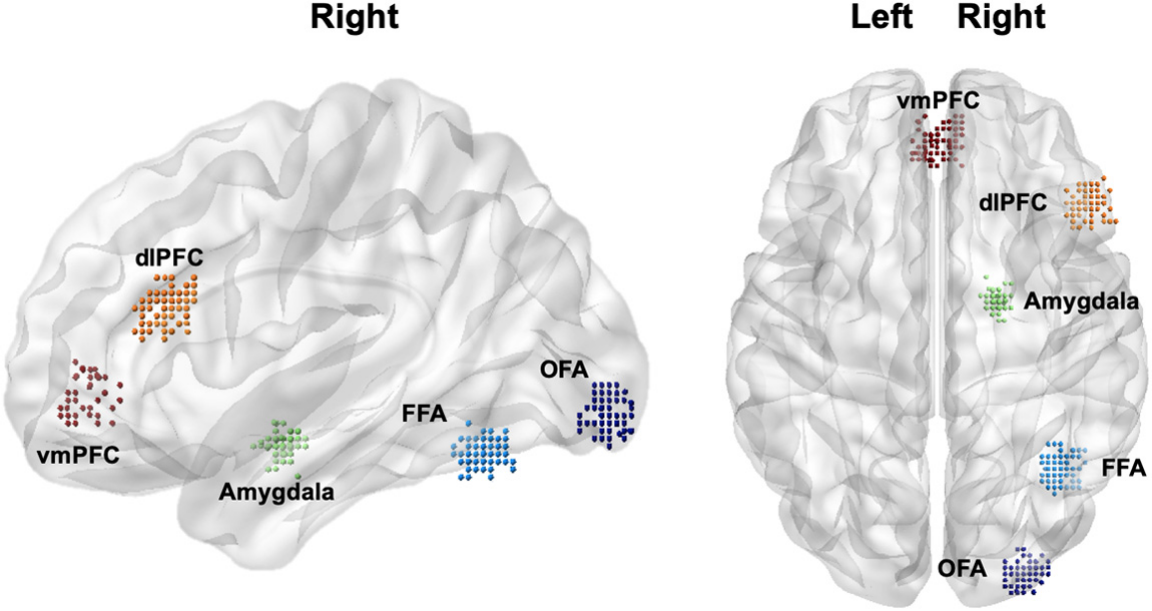
Distribution of the center coordinates of the VOIs for each subject. Render visualized using BrainNet Viewer (Xia et al., 2013). OFA = occipital face area; FFA = fusiform face area; dlPFC = dorsolateral prefrontal cortex; vmPFC = ventromedial prefrontal cortex.

Constructing a candidate model space relies on extracting summaries of time series from different brain regions at an individual subject level. Our chosen volumes of interest (VOIs) were informed by anatomic network models of emotional face processing (Fairhall and Ishai, 2007; Palermo and Rhodes, 2007; Dima et al., 2011) and included the OFA, FFA, amygdala, dlPFC, and vmPFC (for group-level coordinates, see Table 1). The specified coordinates for each region were informed by the group-level GLM results and were restricted to the right hemisphere to allow for the exclusion of fewer participants because of inadequate activation. While both the left and right hemispheres were activated during this task, greater activity has previously been observed in the right hemisphere for this task (Hariri et al., 2002), including more consistent right-sided activity at an individual subject level during the processing affective facial stimuli (Fairhall and Ishai, 2007). The OFA, FFA, amygdala, and dlPFC were defined by the sad and fearful faces > shapes contrast, while the vmPFC was defined by the inverse of this contrast (Harrison et al., 2011). For each participant, the center coordinates of these VOIs were dependent on their subject-specific local maxima of these regions; these were required to be within 8 mm from the group-level peak (for the resulting distribution of individual coordinates, see Fig. 1). The time series for each VOI was adjusted using an *F*-contrast, thereby mean-correcting these values. As per recently published guidelines, we extracted the principal eigenvariate for each of these regions, calculated using all voxels (at a threshold of *p *<* *0.05, uncorrected) within a sphere with a radius of 4 mm from the VOI’s center (Zeidman et al., 2019). If individuals had inadequate activation of all VOIs this threshold was lowered further, up to a threshold of *p *<* *0.5. As a result, of the 92 participants that underwent analysis, three participants were excluded because of inadequate regional activity.

**Table 1:** Significant activation and deactivation associated with face processing

Brain region	BA	Coordinates			Cluster size (2-mm^3^ voxels)	*t* value
		*x*	*y*	*z*		
Faces > shapes						
Inferior occipital gyrus (OFA)	19	28	−92	−8	14854	21.70
		−22	−98	−2		20.44
Fusiform gyrus (FFA)	37	40	−60	−18		19.70
		−38	−54	−20		15.89
Superior temporal sulcus	39/37/22	50	−46	12		8.15
Dorsal midbrain	NA	24	−32	−2	4653	19.93
		−20	−34	−2		13.78
		8	−34	−2		16.07
		−8	−34	−4		11.65
Amygdala	53	20	−6	−16		15.67
		−18	−8	−16		11.36
Thalamus	50	8	−14	8		12.14
		−8	−16	8		8.92
Inferior frontal gyrus	44	42	8	30	2443	13.46
Middle frontal gyrus (dlPFC)	9	50	26	20		12.60
Supplementary motor cortex	6	40	0	48		8.54
Frontal eye fields	8	2	14	46	810	12.50
Inferior frontal gyrus	44	−38	8	26	1959	11.99
Middle frontal gyrus	9	−46	14	24		11.74
Supplementary motor cortex	6	−40	0	42		9.07
Midcingulate	24	6	2	26	63	9.55
Precuneus	31	4	−60	32	697	9.47
		0	−58	40		9.31
Anterior insular	13	−32	22	−4	435	9.47
		38	22	−4	208	8.35
Shapes > faces						
Inferior parietal lobule	40	−56	−32	28	550	8.99
		58	−30	26	732	7.54
Superior visual association cortex	18	16	−86	20	77	8.45
		−16	−86	26	71	7.12
Dorsal posterior cingulate cortex	31	−6	−28	42	540	7.80
		8	−30	44		7.00
Ventral posterior cingulate cortex	23	−12	−58	14	103	7.82
Lingual gyrus	19	−28	−46	−10	122	7.42
		28	−46	−8	116	7.78
Ventromedial prefrontal cortex (vmPFC)	32	2	48	−4	238	6.21
Inferior parietal cortex	7	18	−50	56	34	5.73

#### Model specification

**Figure 2.**
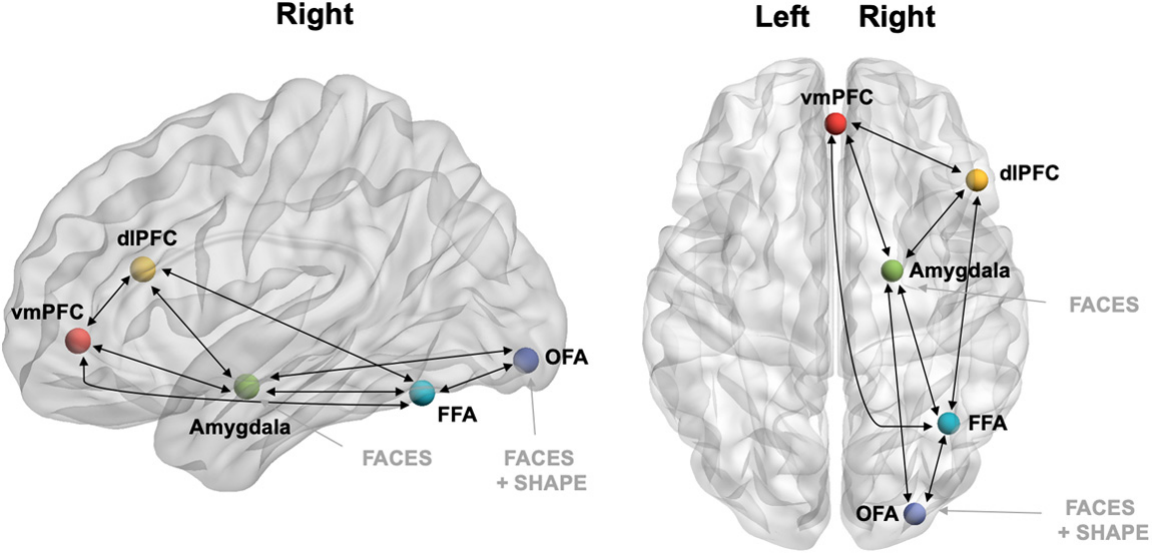
Model of intrinsic connections (black) and extrinsic input (gray) specified in our DCM analysis. Render visualized using BrainNet Viewer (Xia et al., 2013). OFA = occipital face area; FFA = fusiform face area; dlPFC = dorsolateral prefrontal cortex; vmPFC = ventromedial prefrontal cortex.

**Figure 3.**
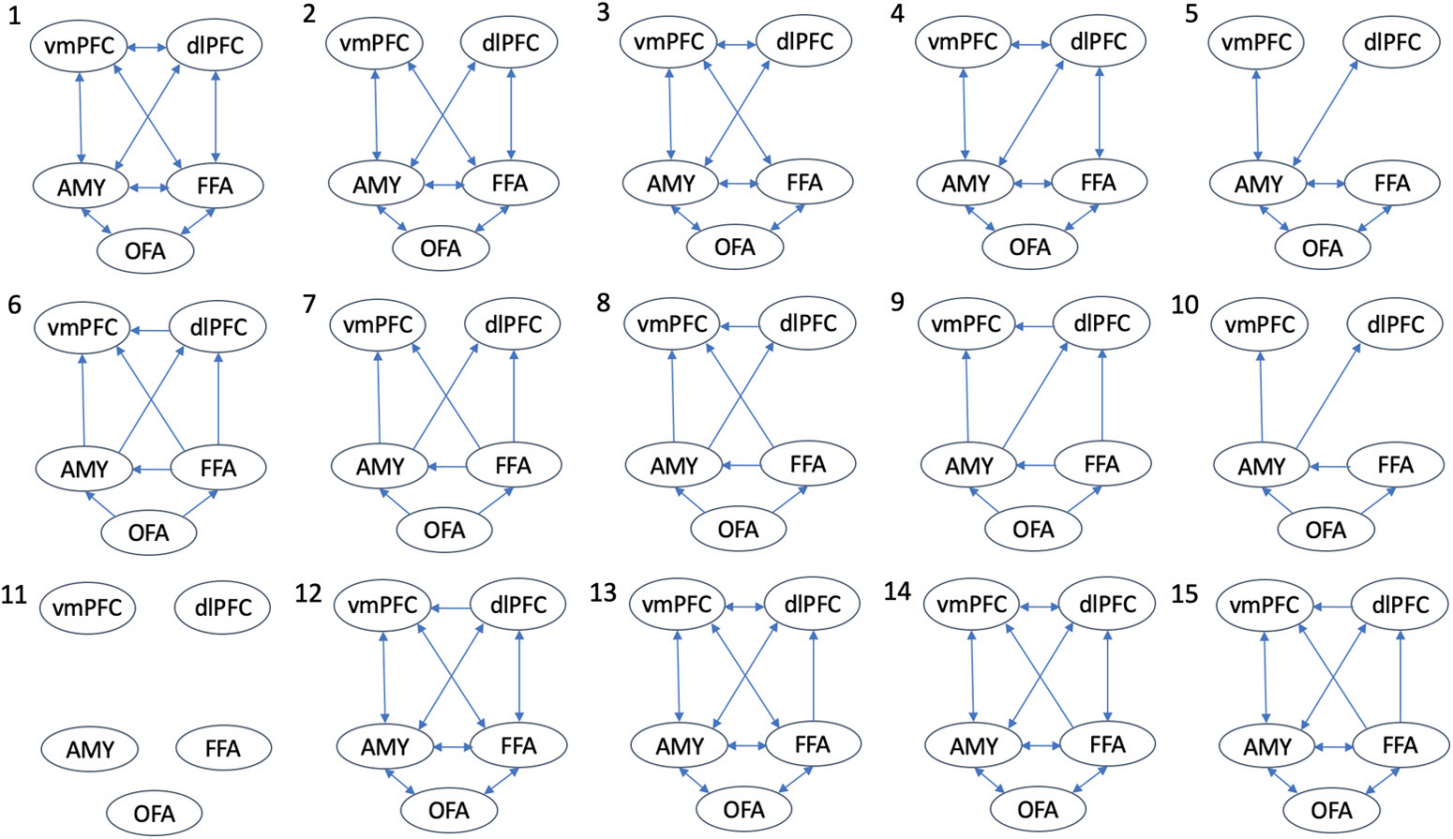
Candidate model space detailing which connections are modulated in each model.

The candidate model space was specified using DCM12.5. The intrinsic connectivity was defined with bidirectional connections between the FFA and OFA, amygdala, dlPFC and vmPFC, between the amygdala and OFA, dlPFC and vmPFC, and between the dlPFC and vmPFC (Fig. 2). This configuration was informed by previous studies investigating the interaction between these regions (Dima et al., 2011; Herrington et al., 2011; Willinger et al., 2019). Notably, while there are minimal direct anatomic connections from the amygdala to dlPFC, this interaction was modeled to account for indirect connections through intermediating regions (Ray and Zald, 2012). Direct external input into the network was modeled using both the effect of all stimuli (shape + fearful + sad) into the OFA and overall negative facial expression (fearful + sad) into amygdala, or input into these regions separately (Diwadkar et al., 2012; Vai et al., 2016). As in previous studies, the amygdala was included as an input region because of the direct influence of the subcortical visual pathway on this area, which is particularly important in fearful expression processing (Phelps and LeDoux, 2005; McFadyen et al., 2019). The input matrix was not mean centered, and as such, the intrinsic connectivity represents unmodeled implicit baseline (Zeidman et al., 2019). Modulation to these intrinsic connections because of shape matching was specified for all connections to establish an active baseline for comparison with the emotional face modulations. Modulations because of fearful or sad facial expression processing, were specified as 15 unique modulation models for each modulation type (see Fig. 3). The combination of modulation and input types resulted in a total of 675 candidate models for each subject (e.g., 15 × 15 × 3), which were grouped into three families of 225 models dependent on the direct input (OFA and amygdala, only OFA, or only amygdala). Model 1 consisted of bidirectional modulation between all VOIs, while models 2, 3, and 4 removed the modulations between the vmPFC and dlPFC, FFA and dlPFC, and FFA and vmPFC, respectively. Model 5 removed all three sets of these modulations. As the existing literature has demonstrated strong evidence for modulation from the core face processing regions to the amygdala and from the amygdala to prefrontal regions (Dima et al., 2011; Herrington et al., 2011; Diwadkar et al., 2012; Vai et al., 2016; Willinger et al., 2019), these models represented alterative interactions which may also contribute to explaining the data. Models 6−10 were feedforward alone versions of models 1−5, while model 11 was a null model with no modulation. Finally, models 12–15 were deviations of models 2–5; however, rather than removing these connections, they were feedforward only.

#### Estimation and inference

We estimated the full model for each participant then deployed Bayesian model reduction for subsequent nested models, thus reducing the computational demands of our large candidate model space (Friston et al., 2003, 2016). We used random-effects Bayesian model selection (RFX BMS), thus allowing for different subjects’ data being optimally explained by different model structures (Stephan et al., 2010). Moreover, Bayesian model averaging (BMA) was used to overcome potential uncertainty concerning model structure (Penny et al., 2010). BMA averages the strength of parameters across different models while weighting these parameters by the posterior probability of their respective models. We then extracted each subjects’ parameter estimates for all intrinsic, modulatory and direct input parameters. The statistical significance of these parameters was determined by one-sample *t* tests in SPSS version 24 (IBMCorp.), which were then false discovery rate corrected for multiple comparisons (Benjamini and Hochberg, 1995). Differences between connectivity strengths were compared through the use of repeated measures ANOVAs. Associations between sad and fearful associated connectivity were assessed through Pearson correlations.

### Correlations between connectivity, behavior, and demographic measures

We further conducted Pearson and Spearman correlations, dependent on variable distributions, between the total connectivity of parameters of interest (amygdala to dlPFC, dlPFC to vmPFC and vmPFC to amygdala) and age, accuracy and the RT to the fearful and sad face matching conditions (correct responses only).

## Results

### Behavioral results

Participants’ mean RTs were found to be significantly different between each of the condition types (repeated measures ANOVA: *F*_(1.34,122.2)_ = 669.38, *p *<* *0.001). *Post hoc* testing demonstrated significantly faster RTs for the shape matching condition compared with both the sad and fearful conditions (both *p *<* *0.001), and a significantly faster RT for sad compared with fearful (*t*_(91)_ = −2.78, *p *=* *0.02; see Table 2). Similarly, response accuracy was significantly different between conditions (Friedman test: χ^2^(2) = 38.79, *p *<* *0.001). *Post hoc* testing demonstrated significantly lower accuracy in responses to sad faces versus both shapes and fearful faces (Wilcoxon signed-rank tests: both *p *<* *0.001, also see Table 2).

**Table 2:** Participants’ behavioral responses for the shape matching and two gender matching conditions

Condition	Mean (SD)	Mean difference (SD)	
		With sad	With fearful
Shape matching			
RT (s)	0.77 (0.17)	0.50 (0.2)**	0.52 (0.2)**
% of correct response	97.66 (3.3)	2.1 (4)	0.12 (34)
Gender matching: sad facial expression			
RT (s)	1.27 (0.22)	-	0.02 (0.08)
% of correct response	95.53(3.0)	-	2.2 (3.4)**
Gender matching: fearful facial expression			
RT (s)	1.29 (0.22)	-	-
% of correct response	97.75 (2.3)	-	-

* Significant at *p* < 0.05, ** significant at *p* < 0.001.

### Mapping brain activation and deactivation responses to sad and fearful faces

**Figure 4.**
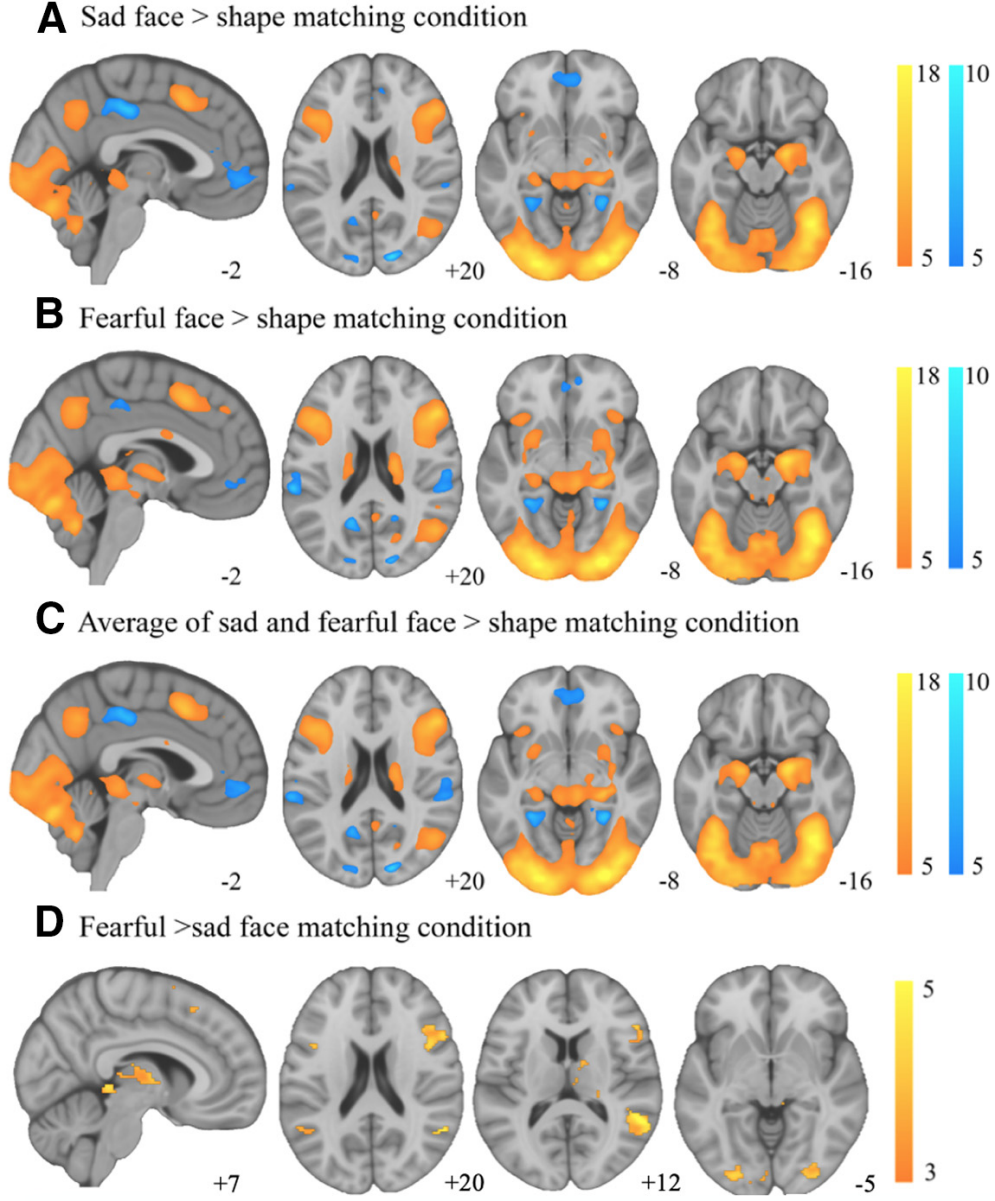
Brain activation (warm) and deactivation (cool) during the emotional face and shape matching conditions. ***A***, Sad faces > shapes. ***B***, Fearful faces > shapes. ***C***, Both faces > shapes (*p*_FWE_ < 0.05). Also shown is the comparison of fearful and sad face matching conditions (***D***). Greater activations for fearful compared with sad faces shown in warm (*p *<* *0.001, uncorrected; cluster-wise correction, *p*_FWE_ < 0.05). No significant deactivation for sad faces compared with fearful were observed.

As depicted in Figure 4*A*,*B*, both the sad and fearful face processing conditions were associated with significant activation of the face processing network, including the inferior occipital gyri, fusiform gyrus, superior temporal sulcus, amygdala, dorsal midbrain, middle frontal gyrus, supplementary motor area, and dorsomedial thalamus. Regions of significant deactivation included the vmPFC, dorsal posterior cingulate and posterior parahippocampal cortices. Figure 4*C* depicts the overall results of emotional face versus shape processing. A full list of all significant regions for this contrast are depicted in Table 1.

When directly comparing the two face conditions, no significant differences were observed for either the magnitude of activation or deactivation (*p*_FWE_ < 0.05). When adopting a more lenient threshold (*p *<* *0.001, uncorrected) and using a *p*_FWE_ < 0.05 cluster-wise correction, greater activation of the inferior occipital gyri, fusiform gyrus, superior temporal sulcus, middle frontal gyrus, dorsal midbrain, and thalamus were observed in response to fearful faces compared with sad faces (Fig. 4*D*). All regions with significant differences in activity between the two face conditions are depicted in Table 3.

**Table 3:** Significant differences in the activations associated processing fearful and sad facial expressions

Brain region	BA	Coordinates			Cluster size (2-mm^3^ voxels)	*t* value
		*x*	*y*	*z*		
Fear > sad						
Superior temporal sulcus	39/37/22	56	−48	12	829	5.21
Inferior occipital/ fusiform gyrus	19/37	−32	−74	−22	898	5.15
		38	−70	−12	579	4.55
Dorsal midbrain/thalamus	36/50	8	−36	0	663	4.91
Middle frontal gyrus (dlPFC)	44	54	18	22	1105	4.70
Superior frontal gyrus	8	−8	32	44	262	4.42
		4	18	70	167	3.05
Lateral precuneus	7	28	−70	32	159	4.39
Superior parietal lobule	39	−50	−62	28	235	4.38

### DCM results

**Figure 5.**
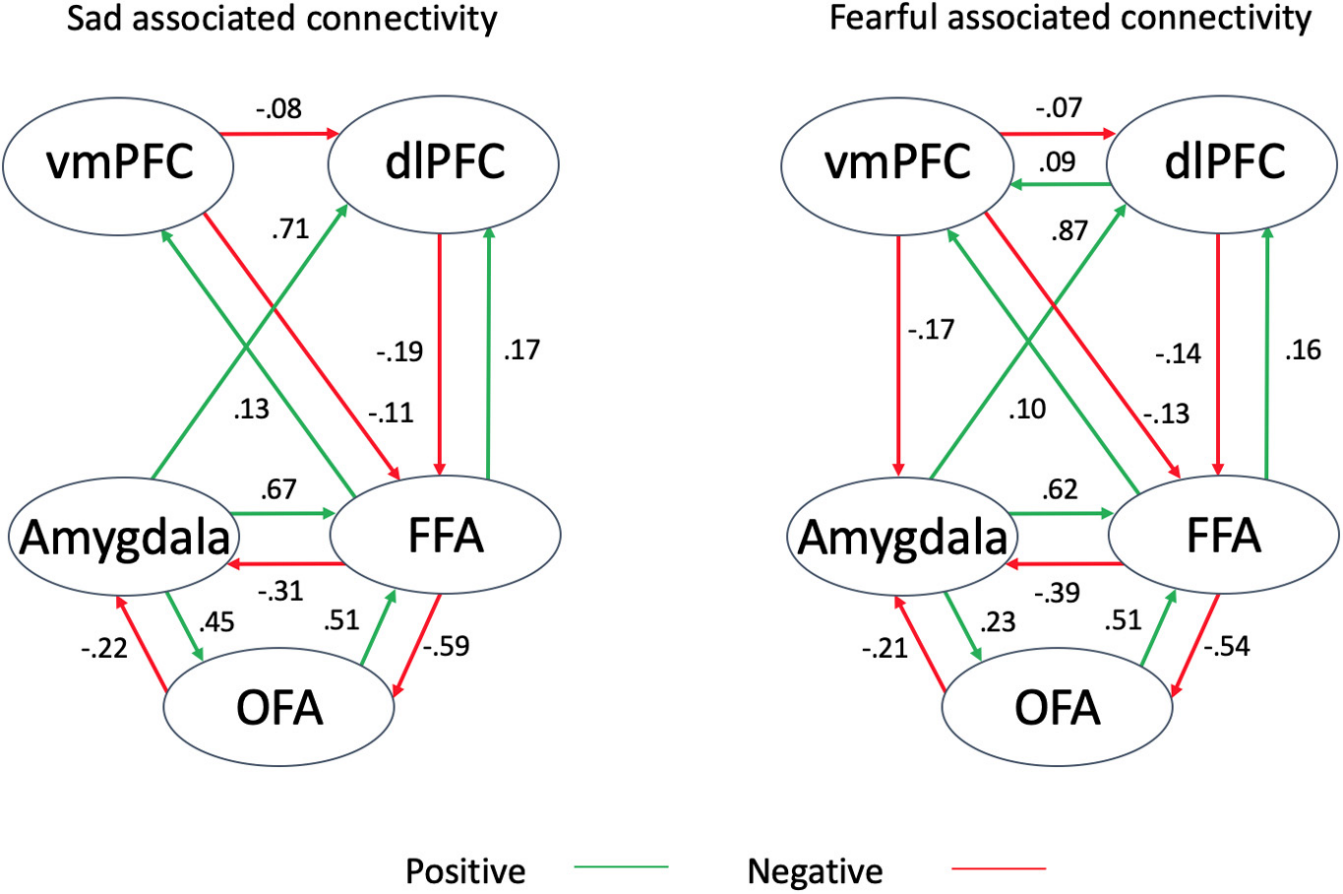
The total connectivity (intrinsic + modulation) of the face processing network associated with sad and fearful facial expression processing. Positive connectivity shown in green, negative connectivity shown in red. OFA = occipital face area; FFA = fusiform face area; dlPFC = dorsolateral prefrontal cortex; vmPFC = ventromedial prefrontal cortex.

To assess the successfulness of model inversion we examined the percentage variance explained using the *spm_dcm_fmri_check* function. This revealed an average explained variance of 55% (SD 15%) across all subjects, suggesting that the data contains useful information relating to our experimental effects (Zeidman et al., 2019). To determine whether the data were better explained by direct inputs (the effect of all faces) entering the system at either the OFA, amygdala or both regions, three families compared these families of models using RFX BMS. This revealed that models with direct inputs into both the OFA and amygdala outperformed both of these inputs individually (expected posterior probability: 0.77, exceedance probability: 1.00). Within this family, model 5 was determined to be the model with the most evidence or “winning model” (expected posterior probability: 0.18, exceedance probability: 0.40). This model indicated that the main influence of both fearful and sad face processing was on bidirectional connections between the OFA and FFA and between the amygdala and vmPFC, FFA, and dlPFC. After applying BMA over all models within the family winning model (direct input to OFA and amygdala), the pathway detailed in model 5 had been largely been conserved, with the addition of the forward only parameters detailed in model 15 (Fig. 5). Significant intrinsic and modulatory connections are reported in Table 4.

**Table 4:** Mean and SD of each parameter estimate for the intrinsic connectivity and their shape, sad, and fearful associated modulations

Connection	Intrinsic connectivity	Shape modulation	Sad modulation	Fearful modulation
	Mean (SD)	Mean (SD)	Mean (SD)	Mean (SD)
OFA→FFA	0.01 (0.20)	−0.04 (0.44)	0.50 (0.43)**	0.50 (0.43)**
OFA→amygdala	−0.05 (0.14)*	0.15 (0.33)**	−0.17 (0.46)*	−0.16 (0.36)**
FFA→OFA	−0.37 (0.34)**	0.00 (0.61)	−0.22 (0.64)*	−0.17 (0.65)*
FFA→amygdala	−0.03 (0.14)*	0.03 (0.28)	−0.28 (0.42)**	−0.36 (0.50)**
FFA→dlPFC	−0.09 (0.24)*	0.07 (0.40)	0.26 (0.29)**	0.25 (0.31)**
FFA→vmPFC	0.01 (0.15)	−0.06 (0.34)	0.12 (0.27)**	0.09 (0.22)*
Amygdala→OFA	0.00 (0.30)	−0.14 (0.54)	0.45 (0.75)**	0.23 (0.70)*
Amygdala→FFA	0.17 (0.16)**	0.14 (0.28)**	0.50 (0.60)**	0.45 (0.64)**
Amygdala→dlPFC	0.14 (0.18)**	0.16 (0.41)*	0.58 (0.52)**	0.73 (0.58)**
Amygdala→vmPFC	−0.01 (0.18)	−0.08 (0.36)	0.09 (0.48)	0.05 (0.55)
dlPFC→FFA	−0.06 (0.18)*	0.04 (0.29)	−0.13 (0.34)*	−0.07 (0.31)*
dlPFC→amygdala	0.02 (0.14)	0.02 (0.21)	0.03 (0.51)	−0.05 (0.41)
dlPFC→vmPFC	−0.01 (0.17)	0.01 (0.32)	0.03 (0.28)	0.10 (0.33)*
vmPFC→FFA	−0.07 (0.17)**	−0.04 (0.36)	−0.04 (0.20)	−0.06 (0.23)*
vmPFC→amygdala	−0.03 (0.14)	−0.04 (0.20)	−0.08 (0.50)	−0.14 (0.48)**
vmPFC→dlPFC	−0.07 (0.19)*	0.00 (0.31)	−0.01 (0.14)	0.01 (0.07)
Direct input				
OFA	-	−0.11 (0.23)**	0.78 (0.60)**	80 (0.54)**
Amygdala	-	-	0.45 (0.38)**	0.55 (0.46)**

OFA = occipital face area; FFA = fusiform face area; dlPFC = dorsolateral prefrontal cortex; vmPFC = ventromedial prefrontal cortex.

* Significant at *p* < 0.05, ** significant at *p* < 0.001.

### Differences between the connections

The coupling of parameters (regions) in DCM is measured in Hertz and represents the rates of change in activity between regions. To determine the overall connectivity between these regions the additive effects of the intrinsic and modulatory parameters for each of these connections were used in these analyses.

**Figure 6.**
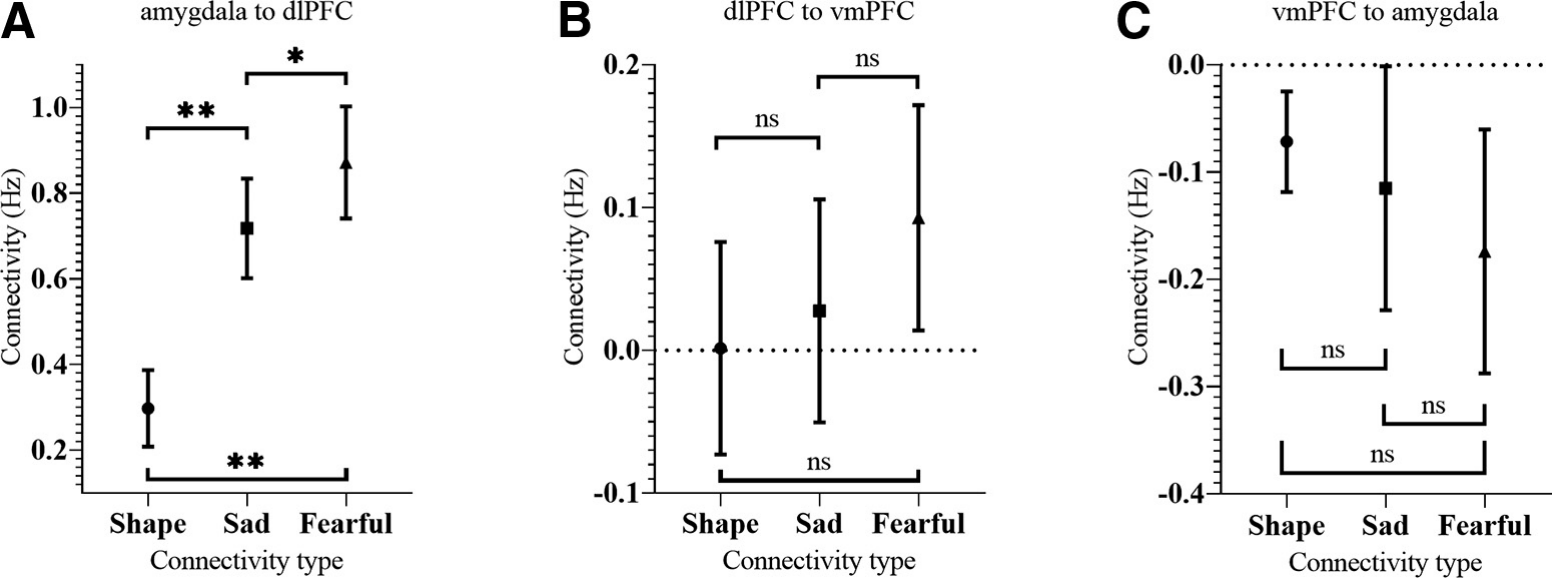
Differences between shape matching, and sad and fearful face processing conditions in the average amount of connectivity from the amygdala to dlPFC (***A***), from the dlPFC to vmPFC (***B***), and from the vmPFC to amygdala (***C***). Error bars indicate 95% CI. *Significant at *p* < 0.05, **significant at *p* < 0.001, ns: not significant.

Participants’ connectivity between the amygdala and dlPFC were found to be significantly different between the condition types (repeated measures ANOVA: *F*_(1.71,150.39)_ = 37.86, *p* < 0.001). *Post hoc* testing revealed that compared with the shape related connectivity, both sad and fearful expression processing were significantly greater by an average of 0.42 and 0.58 Hz, respectively (both *p *<* *0.001). The connectivity from the amygdala to dlPFC was also significantly greater during fearful compared with sad expression procession, by an average of 0.16 Hz (*p *=* *0.004). Therefore, during fearful compared with sad face processing, increased amygdala activity resulted in greater increases to dlPFC activity (Fig. 6*A*). There was no difference in the connectivity types from the dlPFC to vmPFC nor from the vmPFC to amygdala (repeated measures ANOVA: *F*_(2,176)_ = 2.22, *p *=* *0.11, and *F*_(1.72,151.15)_ = 1.40, *p *=* *0.25; Fig. 6*B*,*C*). Interestingly, while sad and fearful associated connectivity from the amygdala to dlPFC and from the dlPFC to vmPFC were significantly correlated with one another (*r *=* *0.65, *p *<* *0.001 and *r *=* *0.42, *p *<* *0.001, respectively), this was not so for vmPFC to amygdala connectivity (*r *=* *0.20, *p *=* *0.06).

### Brain and behavioral relationships

Correlations between connectivity parameters of interest and participants’ RT, accuracy and age are found in Table 5. No significant associations between connectivity parameters and these measures were found.

**Table 5:** Correlations between total connectivity (intrinsic + modulatory) for parameters of interest and age, RT, and accuracy

Connectivity	Age	Respective RT	Respective % correct
Sad associated connectivity			
Amygdala to dlPFC	−0.12	−0.17	−0.06
dlPFC to vmPFC	0.04	−0.19	−0.14
vmPFC to amygdala	0.15	0.09	0.15
Fear associated connectivity			
Amygdala to dlPFC	−16	0.11	0.04
dlPFC to vmPFC	−10	−0.12	0.00
vmPFC to amygdala	0.10	0.20	0.11

No correlations had a *p* < 0.05.

## Discussion

This study has examined the effective connectivity of the face processing network in response to negatively valenced emotional faces, in a large sample of adolescents and young adults. We found evidence to support our first hypothesis, as there was significant positive connectivity from the amygdala to dlPFC under both sad and fearful face processing. Our second hypothesis was partially supported, as significant negative connectivity from the vmPFC to amygdala was only observed for fearful face processing. Additionally, we found a significant difference between the fearful and sad associated connectivity from the amygdala to dlPFC. Overall, the pattern of connectivity observed here is generally consistent with previous investigations of implicit processing of negative expressions, particularly those findings concerning the connectivity from the amygdala to dlPFC and vmPFC to amygdala (Vai et al., 2015; Willinger et al., 2019). While we did not observe the significant modulation from the dlPFC to amygdala reported in the study by Vai et al. (2015), this was likely because of their indirect interaction via the vmPFC in our model.

### Activity of the face processing network and behavioral differences

Both of the emotional face processing conditions evoked robust overlapping patterns of activation in the core and extended face processing systems. Consistent with previous studies, these regions comprised early visual processing areas including the inferior occipital and fusiform gyri as well as the right superior temporal sulcus (Kleinhans et al., 2011; Zhen et al., 2013). We also observed common activation in the amygdala, supplementary motor cortex, and middle frontal gyrus, together with deactivation of the vmPFC, posterior cingulate cortex and inferior parietal lobule (Harrison et al., 2011). These findings support a wealth of literature detailing the functional neuroanatomy of face processing (Haist and Anzures, 2017). In addition to this pattern of strong common activity, we observed some evidence for altered activation of core and extended regions under fearful compared with sad face processing. This included increased activation of the inferior occipital gyri, fusiform gyri, right superior temporal sulcus, and right middle frontal gyrus. This increased activity has been suggested by previous work to be because of the higher salience and attention capturing nature of these fearful stimuli (Hedger et al., 2015), which was further explored through our connectivity analysis.

### Connectivity from the amygdala to dlPFC and salience detection

We observed significant positive connectivity from the amygdala to dlPFC under all three conditions. Significantly greater connectivity was observed for both the sad and fearful expression processing conditions compared with the shape matching condition. Additionally, we found evidence for greater connectivity in response to processing fearful compared with sad faces, which we interpret as resulting from the greater salience and arousal-evoking nature of fearful facial expressions (Adolphs, 2013).

Throughout behavioral and neuroimaging work, emotional stimuli have been reported to have greater salience than neutral stimuli (Vuilleumier, 2005). Moreover, fearful facial expressions have been reported to be both more intense and attention capturing than sad expressions (Langner et al., 2010; Hedger et al., 2015; Lin et al., 2016). The ordinal nature of these effects, as well as the connectivity between the amygdala and dlPFC between conditions, mirrors the arousal-driven amygdala response reported in previous studies (Lin et al., 2020). Researchers have hypothesized that features associated with fearful expressions, such as increased widened eyes, may facilitate their promotion to conscious perception (Hedger et al., 2015; Barrett, 2018). As the amygdala has been shown to preferentially respond to such features (Whalen et al., 2004), it is likely that the increased influence of the amygdala on the dlPFC represents a neural mechanism responsible for orientating conscious attention to salient stimuli (Frank and Sabatinelli, 2012). These findings and our own are consistent with the threat processing model of LeDoux and Pine (2016), which proposes that interactions between subcortical regions, including the amygdala, and the lateral prefrontal cortices are necessary to generate conscious labeling and awareness of feelings. Further research will be necessary to determine whether this effect can be seen for positively valenced facial expressions and how the interaction of valence and arousal alters this modulation.

As the amygdala transmits minimal direct output to the dlPFC, its capacity to influence dlPFC activity presumably occurs through mediatory regions, including the anterior cingulate, vmPFC and ventrolateral prefrontal cortices (Bracht et al., 2009; Ray and Zald, 2012; Vossel et al., 2014). Mechanistically, medial and lateral pathways from the amygdala traverse the inferior thalamic peduncle (interacting with the anterior cingulate cortex) and external capsule, respectively, to interact with the dlPFC (Bracht et al., 2009). Thus, the interaction between these regions is expected to be more complex than framed within this analysis. This may, in part, explain the lack of associations between the amygdala to dlPFC connectivity and RT or accuracy, as other regions associated with salience processing, including the anterior insula, cingulate, and caudate may also contribute to this process (Menon and Uddin, 2010; Damiani et al., 2020).

### The regulatory role of the vmPFC on the amygdala during emotional face processing

Fearful face processing was associated with negative connectivity from the vmPFC to amygdala, which is broadly consistent with previous findings (Sladky et al., 2015; Willinger et al., 2019). This supports other observations that processing these stimuli leads to a regulatory effect on the amygdala (Braunstein et al., 2017). Notably, however, there was no significant modulation during sad face processing, nor a significant difference in this connectivity between the fearful and either the shape or sad conditions.

As previously stated the vmPFC is a component of the default mode network and demonstrates consistent deactivation during cognitively-demanding tasks (Raichle et al., 2001). The magnitude of the vmPFC’s suppression is generally considered to be a correlate of increased task difficulty (Harrison et al., 2011). However, the observed increased in positive modulation from the dlPFC to vmPFC during the processing of fearful expressions would result in less suppression of the vmPFC. This suggests that there may be two opposing influences affecting vmPFC activity: the evaluation of emotional stimuli and cognitive difficulty of this process (Hiser and Koenigs, 2018; Satpute and Lindquist, 2019). Conceptually, this is consistent with recent models which have argued that the vmPFC is important for integrating valence information and contextual knowledge during attentional processes to construct affective meaning (Roy et al., 2012; Winecoff et al., 2013; Viviani, 2014; Winker et al., 2019). As such, regions of the dorsal attentional network including the frontal eye fields have been hypothesized to enable selection of stimuli based on internal goals and drive vmPFC deactivation (Corbetta et al., 2008; Viviani, 2014). Conversely, ventral attentional areas including the dlPFC detect salient, particularly unattended, stimuli within the environment and result in vmPFC activation (Corbetta et al., 2008; Viviani, 2014). These effects are likely to contribute to the large heterogeneity and lack of correlation between fearful and sad associated connectivity from the vmPFC to amygdala, as the amount of regulation that the vmPFC exhibits on the amygdala may not directly reflect the features of the expressions being processed, but individuals’ constructed affective interpretations (Skerry and Saxe, 2015; Barrett, 2017; Satpute and Lindquist, 2019). Further work will be necessary to understand how the temporal dynamics between the dorsal and ventral attentional areas alter vmPFC activity and its regulatory influence over the amygdala.

### Changes associated with the core face processing system

The connectivity between regions of the core and extended systems appears to mostly reflect findings from previous studies. While few studies have modeled expression associated modulation of connectivity in the core system, those that have illustrate greater positive modulation between the OFA and FFA during emotional face processing (Fairhall and Ishai, 2007; Li et al., 2010; Frässle et al., 2016). Unexpectedly, we observed a negative modulation between the FFA and amygdala. While some implicit processing studies have reported negative intrinsic connectivity, few have reported the modulation of these connections (Vai et al., 2016; Willinger et al., 2019). Studies investigating explicit facial expression processing have observed a positive modulation of the connectivity from the FFA to amygdala (Herrington et al., 2011). Further research is required to clarify whether the type of face processing task truly alters these connections.

### Limitations

While this study contains strengths, including its large sample size compared with previous investigations, it is not without limitations. No neutral or positive valenced facial stimuli were used in this task. While this decision was made to maximize task efficiency, examining a wider range of emotional expressions with varying levels of arousal and valence would enhance our ability to disentangle these functions. A contiguous acquisition scheme would have been more advantageous for minimizing the mixing of time series from slices that were acquired at different times and may have allowed for signal extraction which more accurately reflected the underlying neural responses (Stephan et al., 2010) While beyond the scope of our current model, both the anterior insular and cingulate may have also been of interest because of their known involvement in emotion processing. Though they were identified through GLM analysis, they demonstrated insufficient individual activation to be extracted for DCM analysis.

In conclusion, this study expands on our understanding of the functional dynamics implicated in emotional face processing. Specifically, this research examined how interactions between the amygdala, dlPFC, and vmPFC are changed because of processing fearful and sad emotional expressions. Notably, connections within this circuit appear to be greater overall for fearful face processing. Although the connectivity from the amygdala to dlPFC likely represents the processing of similar features in fearful and sad faces, the connectivity from the vmPFC to amygdala may be responding to a higher order conceptualization of emotion. This work contributes toward building more refined models of the brain network dynamics implicated in processing emotional expressions. In turn, these models may inform the ongoing characterization of emotional brain disorders, in which, impairments to emotional face processing are common.

## Synthesis

Reviewing Editor: Karen Davis, Krembil Research Institute and University of Toronto Decisions are customarily a result of the Reviewing Editor and the peer reviewers coming together and discussing their recommendations until a consensus is reached. When revisions are invited, a fact-based synthesis statement explaining their decision and outlining what is needed to prepare a revision will be listed below. The following reviewer(s) agreed to reveal their identity: Franco Cauda.

This paper investigates the integration of information into a distributed network of 5 areas (occipital and fusiform face areas, amygdala, dorsolateral (dlPFC) and ventromedial prefrontal cortices (vmPFC))

during a face processing task. To do this, the authors investigated the effective connectivity of face processing using DCM approach during emotional paradigm (sad and fearful expressions) in 98 healthy adolescents and young adults. The paper is well written and the methodological approach is reasonable and well executed and the descriptions of the results are clear. The results show that the connectivity from amygdala and dlPFC appears to be related to dimensional features which differ between these expressions. This article investigates a topic of interest that in fact has been studied by several other papers. Furthermore,the manuscript seems to be lacking important logical and rational steps that do not permit the reader to have a clear idea of the contribution of this paper to the current literature.

Major Comments:

1. The introduction does not address in sufficient depth the implications of the pre-existing literature and does not sufficiently anticipate the arguments made during the discussion.

2. As for the fMRI acquisition scheme, the authors acquired data using Interleaved acquisition while Stephan, Friston and al (Stephan et al., 2010) strongly suggest to use contiguous acquisition scheme. The authors should provide an explanation of this point.

3. The authors choose to investigate only the right hemisphere due to its role in face processing as stated in "The specified coordinates for each region were informed by the group-level GLM results and were restricted to the right hemisphere, given existing evidence of right-side dominance of this network (Sorger et al., 2007; Rui de Moraes et al., 2014), ...". The right and the left network may not be dissimilar by dominance instead are more related, for example, to process different aspect of the stimuli (Rui de Moraes et al., 2014) and the amygdala itself seems to be more involved during fear processing on the left side instead of the right one (Allen, Bobnar, & Kolber, 2020). The authors should better justify this lateralization choice.

4. The authors used shapes condition as a high-level baseline condition. This condition could be tested in DCM model estimation to look at what are the interactions between nodes during "baseline" condition. This may help to identify a significant change in effectivity connectivity caused by emotional content instead of due to a "general" visual processing.

5. "Both sad and fearful face processing were associated with negative connectivity from the vmPFC to amygdala, which is broadly consistent with previous findings (Sladky et al., 2015; Willinger et al., 2019). Thus, despite the deactivation in vmPFC, its regulatory role over the amygdala appears to be upheld. " While the negative connectivity is confirmed by other studies, the association between deactivation and inhibition is not clear. What is the author’s explanation on this point? I think that need be clarified however is very arduous to interpret this apparent contradiction. This could be explained by the intervention of other areas (such as anterior cingulate cortex) during signal modulation, however it needs to be tested.

6. "Increased cognitive load has also been associated with decreased regulation of the amygdala by the vmPFC (Minkova et al., 2017). The greater difficulty during the processing of sad stimuli may, therefore, contribute to the decreased regulation of the amygdala." Why are sad stimuli more difficult to processing? This sentence needs to be cited and provided the rationale of this claim.

7. Although the study by Willinger et al. (2019) noted significant negative modulation for both positively and negatively valenced expressions, this was overall greater for negative faces. This indicates that the connectivity from the vmPFC to amygdala is influenced by valence, however, our results suggest this is not the only factor." The authors included only negative valence in their analysis, justifying this choice "to maximize task efficiency". However, the rationale beyond this decision seems arbitrary for two main reasons:

i) the idea of choosing sad and fear needs to be explained in greater detail (We chose to focus on fearful and sad facial expressions due to the importance of negative affective processing to models of psychopathology). Apart from clinical application, these two emotions need to be characterized by their arousal-valence and their effect on brain activity. As expected, these two emotions (that mainly differ by the arousal) do not show notable changes in brain activity (the correction approach used in fear > sad was more liberal compared to the others) and in DCM effective connectivity. The difference between fear and sadness needs to be discussed and better justify.

ii) Instead of using two similar valence emotions, the authors could use two opposite valence emotion with similar arousal, such as happiness vs fear, that may help to understand better how face-processing network actually work and their interaction in effective connectivity (Diano et al., 2017; Lettieri et al., 2019)

8. "While we did not observe the significant modulation from the dlPFC to amygdala reported in the study by Vai et al. (2015), this was likely due to their indirect interaction via the vmPFC in our model." "This may, in part, explain the lack of associations between this connectivity and RT or accuracy, as other regions associated with salience processing, including the anterior insula, cingulate and caudate may also contribute to this process (Menon and Uddin, 2010; Damiani et al., 2020)." It is comprehensible the idea of reducing the complexity of the data by using less ROIs during the DCM analysis. However, the authors seem to neglect important regions in emotional processing such as insula, cingulate and subcortical regions such as thalamus that can explain better the findings. The authors should provide a statement and rationale about this decision or analysed the data using more ROIs as founded in sad + fear > shapes fMRI contrast.

9. "For each subject, the coordinates of the VOIs were allowed to differ dependent on their local maxima, with movement constrained to within 8 mm of the group-level peak." The authors should better clarify this sentence. Has each time course been extracted at the subject level (i.e. time course of subject 1 has been deduced from the ROIs based on the subject 1 GLM’s results) or from group level GLM analysis? I think this information is implicit in the next session but it is not clear. If the time course was extracted at the subject level, the authors should include an image of probabilistic displacement of these ROIs.

Minor Comments:

1. An image that synthesizes the results in a more user-friendly and intuitive way could make it easier to read.

2. Please better specify the "matching" phase in the experimental design. Once the face has been shown to the top half of the screen, the two faces presented at the bottom half are they sad/fearful expressions or sad or fearful expression plus neutral face?

3. Please indicate the volume of the cluster size that has been reported in the Table (1). This helps the reader to understand the correct size of a cluster.

4. Figure 1: please specify the left right orientation of the image (should be right-right orientation)

5. Table 2: please add the significant score in the table and not only in the Behavioural results.

BIBLIOGRAPHY

Allen, H. N., Bobnar, H. J., & Kolber, B. J. (2020). Left and right hemispheric lateralization of the amygdala in pain. Prog Neurobiol, 101891. doi:10.1016/j.pneurobio.2020.101891

Diano, M., Tamietto, M., Celeghin, A., Weiskrantz, L., Tatu, M. K., Bagnis, A., . . . Costa, T. (2017). Dynamic Changes in Amygdala Psychophysiological Connectivity Reveal Distinct Neural Networks for Facial Expressions of Basic Emotions. Sci Rep, 7, 45260. doi:10.1038/srep45260

Lettieri, G., Handjaras, G., Ricciardi, E., Leo, A., Papale, P., Betta, M., . . . Cecchetti, L. (2019). Emotionotopy in the human right temporo-parietal cortex. Nat Commun, 10(1), 5568. doi:10.1038/s41467-019-13599-z

Stephan, K. E., Penny, W. D., Moran, R. J., den Ouden, H. E., Daunizeau, J., & Friston, K. J. (2010). Ten simple rules for dynamic causal modeling. Neuroimage, 49(4), 3099-3109. doi:10.1016/j.neuroimage.2009.11.015

## Author Response

Major Comments:

1. The introduction does not address in sufficient depth the implications of the pre-existing literature and does not sufficiently anticipate the arguments made during the discussion.

Response

We have attempted to address this comment by substantially modifying paragraph two and three of the Introduction (page 3). These alterations aimed to clarify the selection of the stimuli and how previous studies have implicated our regions of interest in processing differences in emotional arousal. Through these changes, we feel that the study’s rationale is clearer and that the arguments made during the discussion are better anticipated.

"The amygdala is traditionally implicated in the processing of fearful stimuli, however, meta-analyses have associated amygdala activity with both positively and negatively valenced expressions (Fusar-Poli et al., 2009). As such, the amygdala has been hypothesized to respond to novel and highly salient information (Blackford et al., 2010; Todorov, 2012; Jacob et al., 2014). This is further supported by greater amygdala activity during the processing of fearful compared with sad expressions (Fusar-Poli et al., 2009), two negatively valenced expressions which differ in their arousal ratings (Hedger et al., 2015; Lin et al., 2016). Moreover, increased connectivity between the amygdala and dlPFC has been commonly identified in face processing tasks (Dannlowski et al., 2009; Comte et al., 2016; Haller et al., 2018). Depending on the directionality, the interaction between the dlPFC and amygdala may be important in directing conscious awareness towards and regulating emotional responses to salient emotional stimuli (Dolcos et al., 2006; Banks et al., 2007; Etkin et al., 2015). Despite this, previous research has not examined whether these directional interactions are altered during the processing of facial expressions with differing arousal ratings, such as fearful and sad expressions (Sladky et al., 2015; Vai et al., 2015; Willinger et al., 2019).

The vmPFC has been consistently implicated in the processing of emotional expressions (Heberlein et al., 2008; Hiser and Koenigs, 2018; Yang et al., 2019). The decreased activity observed during implicit emotional processing tasks is consistent with its involvement in the default mode network (Raichle et al., 2001; Harrison et al., 2008; Harrison et al., 2011). Negatively valenced expressions, particularly sad expressions, have demonstrated greater vmPFC deactivation in comparison to happy expressions (Sreenivas et al., 2012). While this suggests that the vmPFC is sensitive to emotional valence, other research proposes it may also respond to emotional arousal (Zhang et al., 2014; Kuniecki et al., 2018). As vmPFC to amygdala connectivity appears crucial in regulating appropriate behavioral responses to emotional stimuli, changes to the valence and arousal of these stimuli likely influences this regulation (Hartley and Phelps, 2010; Milad and Quirk, 2012; Braunstein et al., 2017). A recent study by Willinger et al. (2019) examined the effects of valenced facial expressions on the directional interactions between these regions. Although they highlighted that negatively valenced expressions were associated with greater negative modulation from the vmPFC to amygdala, expressions within the negatively valenced category were not compared. Thus, whether expressions which differ in their arousal differentially alter the connectivity of the extended face processing system lacks clarification."

2. As for the fMRI acquisition scheme, the authors acquired data using Interleaved acquisition while Stephan, Friston and al (Stephan et al., 2010) strongly suggest to use contiguous acquisition scheme. The authors should provide an explanation of this point.

Response

A contiguous acquisition scheme would have been optimal, however, as per Kiebel et al. (2007) and the SPM mailing list (den Ouden, 2012), interleaved data with a TR of 2 seconds and which has been slice timing corrected is sufficiently appropriate for DCM. This has been deployed in several previous DCM studies (Musgrove et al., 2015; Sladky et al., 2015; Davey et al., 2016). Nevertheless, we have acknowledged that the acquisition scheme might have affected the extracted time-series in the limitations.

"A contiguous acquisition scheme would have been more advantageous for minimizing the mixing of time series from slices that were acquired at different times and may have allowed for signal extraction which more accurately reflected the underlying neural responses (Stephan et al., 2010)."

3. The authors choose to investigate only the right hemisphere due to its role in face processing as stated in "The specified coordinates for each region were informed by the group-level GLM results and were restricted to the right hemisphere, given existing evidence of right-side dominance of this network (Sorger et al., 2007; Rui de Moraes et al., 2014), ...". The right and the left network may not be dissimilar by dominance instead are more related, for example, to process different aspect of the stimuli (Rui de Moraes et al., 2014) and the amygdala itself seems to be more involved during fear processing on the left side instead of the right one (Allen, Bobnar, & Kolber, 2020). The authors should better justify this lateralization choice.

Response

We agree that the term dominance is incorrect and that it does not accurately reflect the complexity of the processes occurring. Allen, Bobnar, & Kolber (2020) note that while the left hemisphere appears to show more activity to fearful stimuli, that factors such as being image-related stimuli (Markowitsch, 1998) and unconscious processing (Morris et al., 1998) are both associated with increased right side activity. We have revised this section to clarify our justification in more detail (page 10, paragraph 2).

"Constructing a candidate model space relies on extracting summaries of time-series from different brain regions at an individual subject level. Our chosen volumes of interest (VOIs) were informed by anatomical network models of emotional face processing (Fairhall and Ishai, 2007; Palermo and Rhodes, 2007; Dima et al., 2011) and included the OFA, FFA, amygdala, dlPFC and vmPFC (for group-level coordinates see Table 1). The specified coordinates for each region were informed by the group-level GLM results and were restricted to the right hemisphere to allow for the exclusion of fewer participants due to inadequate activation. While both the left and right hemispheres were activated during this task, greater activity has previously been observed in the right hemisphere for this task (Hariri et al., 2002), including more consistent right-sided activity at an individual subject level during the processing affective facial stimuli (Fairhall and Ishai, 2007). The OFA, FFA, amygdala, and dlPFC were defined by the sad and fearful faces > shapes contrast, while the vmPFC was defined by the inverse of this contrast (Harrison et al., 2011). For each participant, the center coordinates of these VOIs were dependent on their subject specific local maxima of these regions; these were required to be within 8 mm from the group-level peak (for the resulting distribution of individual coordinates see Figure 1)."

4. The authors used shapes condition as a high-level baseline condition. This condition could be tested in DCM model estimation to look at what are the interactions between nodes during "baseline" condition. This may help to identify a significant change in effectivity connectivity caused by emotional content instead of due to a "general" visual processing.

Response

Thank you for the suggestion. We have re-estimated our models with this modulation included and updated our results (page 15, paragraph 3), as well as Table 4 and Figure 5 (below) In short, the relationship from the amygdala to dlPFC remained unchanged, however, there was no longer significant sad associated modulation from the vmPFC to amygdala nor was this modulation different between the three conditions. As such, we have also updated the relevant parts of the discussion (see response 5).

‘Participants’ connectivity between the amygdala and dlPFC were found to be significantly different between the condition types (repeated measures ANOVA: F(1.71, 150.39) = 37.86, P < 0.001). Post hoc testing revealed that compared with the shape related connectivity, both sad and fearful expression processing were significantly greater by an average of .42 and .58 Hz, respectively (both P < 0.001). The connectivity from the amygdala to dlPFC was also significantly greater during fearful compared with sad expression procession, by an average of .16 Hz (P = .004). Therefore, during fearful compared with sad face processing, increased amygdala activity resulted in greater increases to dlPFC activity (Figure 6A). There was no difference in the connectivity types from the dlPFC to vmPFC nor from the vmPFC to amygdala (repeated measures ANOVA: F(2, 176) = 2.22, P = 0.11, and F(1.72, 151.15) = 1.40, P = 0.25; Figure 6B & C). Interestingly, while sad and fearful associated connectivity from the amygdala to dlPFC and from the dlPFC to vmPFC were significantly correlated with one another (r = 0.65, P < 0.001 and r = .42, P < 0.001, respectively), this was not so for vmPFC to amygdala connectivity (r = 0.20, P = 0.06)."

5. "Both sad and fearful face processing were associated with negative connectivity from the vmPFC to amygdala, which is broadly consistent with previous findings (Sladky et al., 2015; Willinger et al., 2019). Thus, despite the deactivation in vmPFC, its regulatory role over the amygdala appears to be upheld. " While the negative connectivity is confirmed by other studies, the association between deactivation and inhibition is not clear. What is the author’s explanation on this point? I think that need be clarified however is very arduous to interpret this apparent contradiction. This could be explained by the intervention of other areas (such as anterior cingulate cortex) during signal modulation, however it needs to be tested.

Response

This section has been revised to incorporate the new results and to further expand upon a likely cause of this effect, which we agree, defies simple explanation. Page 18, paragraph 3, now reads:

"Fearful face processing was associated with negative connectivity from the vmPFC to amygdala, which is broadly consistent with previous findings (Sladky et al., 2015; Willinger et al., 2019). This supports other observations that processing these stimuli leads to a regulatory effect on the amygdala (Braunstein et al., 2017). Notably, however, there was no significant modulation during sad face processing, nor a significant difference in this connectivity between the fearful and either the shape or sad conditions.

As previously stated the vmPFC is a component of the default mode network and demonstrates consistent deactivation during cognitively-demanding tasks (Raichle et al., 2001). The magnitude of the vmPFC’s suppression is generally considered to be a correlate of increased task difficulty (Harrison et al., 2011). However, the observed increased in positive modulation from the dlPFC to vmPFC during the processing of fearful expressions would result in less suppression of the vmPFC. This suggests that there may be two opposing influences affecting vmPFC activity: the evaluation of emotional stimuli and cognitive difficulty of this process (Hiser and Koenigs, 2018; Satpute and Lindquist, 2019). Conceptually, this is consistent with recent models which have argued that the vmPFC is important for integrating valence information and contextual knowledge during attentional processes to construct affective meaning (Roy et al., 2012; Winecoff et al., 2013; Viviani, 2014; Winker et al., 2019). As such, regions of the dorsal attentional network including the frontal eye fields have been hypothesized to enable selection of stimuli based on internal goals and drive vmPFC deactivation (Corbetta et al., 2008; Viviani, 2014). Conversely, ventral attentional areas including the dlPFC detect salient, particularly unattended, stimuli within the environment and result in vmPFC activation (Corbetta et al., 2008; Viviani, 2014). These effects are likely to contribute to the large heterogeneity and lack of correlation between fearful and sad associated connectivity from the vmPFC to amygdala, as the amount of regulation that the vmPFC exhibits on the amygdala may not directly reflect the features of the expressions being processed, but individuals’ constructed affective interpretations (Skerry and Saxe, 2015; Barrett, 2017; Satpute and Lindquist, 2019). Further work will be necessary to understand how the temporal dynamics between the dorsal and ventral attentional areas alter vmPFC activity and its regulatory influence over the amygdala."

6. "Increased cognitive load has also been associated with decreased regulation of the amygdala by the vmPFC (Minkova et al., 2017). The greater difficulty during the processing of sad stimuli may, therefore, contribute to the decreased regulation of the amygdala." Why are sad stimuli more difficult to processing? This sentence needs to be cited and provided the rationale of this claim.

Response

This statement was in reference to the greater percentage of incorrect responses for sad expression processing. However, due to changes in the results from modelling the shape condition this section has been removed and replaced with the paragraph highlighted in response 5.

7. Although the study by Willinger et al. (2019) noted significant negative modulation for both positively and negatively valenced expressions, this was overall greater for negative faces. This indicates that the connectivity from the vmPFC to amygdala is influenced by valence, however, our results suggest this is not the only factor." The authors included only negative valence in their analysis, justifying this choice "to maximize task efficiency". However, the rationale beyond this decision seems arbitrary for two main reasons:

I. the idea of choosing sad and fear needs to be explained in greater detail (We chose to focus on fearful and sad facial expressions due to the importance of negative affective processing to models of psychopathology). Apart from clinical application, these two emotions need to be characterized by their arousal-valence and their effect on brain activity. As expected, these two emotions (that mainly differ by the arousal) do not show notable changes in brain activity (the correction approach used in fear > sad was more liberal compared to the others) and in DCM effective connectivity. The difference between fear and sadness needs to be discussed and better justify.

Response

As highlighted in our response to point 1, we have revised the introduction to improve our justification and address the differences between these expressions (page 3, paragraph 2). This is specifically addressed in the following sections:

"Studies have hypothesized that the amygdala’s role in face processing is to respond to novel and highly salient information (Blackford et al., 2010; Todorov, 2012; Jacob et al., 2014). This is consistent with the finding of greater activity during the processing of fearful compared with sad expressions (Fusar-Poli et al., 2009), as although these expressions are both negatively valenced they differ in ratings of arousal (Hedger et al., 2015; Lin et al., 2016).

"Despite this, previous research examining the directionality of these interactions has not examined whether facial expressions with differing arousal ratings, such as fearful and sad expressions, differently modulate this relationship (Sladky et al., 2015; Vai et al., 2015; Willinger et al., 2019)."

II. Instead of using two similar valence emotions, the authors could use two opposite valence emotion with similar arousal, such as happiness vs fear, that may help to understand better how face-processing network actually work and their interaction in effective connectivity (Diano et al., 2017; Lettieri et al., 2019)

Response

While this suggestion would indeed improve the characterization of how valence alters the face processing network, it is essentially the work done by Willinger et al., 2019. We agree that future research should more closely examine these effects together and compare the interaction of valence and arousal. This has been highlighted within the discussion (page 18, paragraph 1):

"Further research will be necessary to determine whether this effect can be seen for positively valence facial expressions, and how the interaction of valence and arousal alters this modulation."

As well as in our limitations (page 20, paragraph 2):

"...examining a wider range of emotional expressions with varying levels of arousal and valence would enhance our ability to disentangle these functions."

8. "While we did not observe the significant modulation from the dlPFC to amygdala reported in the study by Vai et al. (2015), this was likely due to their indirect interaction via the vmPFC in our model." "This may, in part, explain the lack of associations between this connectivity and RT or accuracy, as other regions associated with salience processing, including the anterior insula, cingulate and caudate may also contribute to this process (Menon and Uddin, 2010; Damiani et al., 2020)." It is comprehensible the idea of reducing the complexity of the data by using less ROIs during the DCM analysis. However, the authors seem to neglect important regions in emotional processing such as insula, cingulate and subcortical regions such as thalamus that can explain better the findings. The authors should provide a statement and rationale about this decision or analysed the data using more ROIs as founded in sad + fear > shapes fMRI contrast.

Response

We agree with the reviewers that both the insula and cingulate are regions of interest when investigating emotional face processing. We had attempted to include these regions, however, they were not consistently activated at an individual subject level to be extracted for a large number of subjects and thus were unable to be feasibly modelled. In addition to the points highlighted above, this is noted in the limitations (page 30, paragraph 3).

"While beyond the scope of our current model, both the anterior insular and cingulate may have also been of interest due to their known involvement in emotion processing. Though they were identified through GLM analysis, they demonstrated insufficient individual activation to be extracted for DCM analysis."

9. "For each subject, the coordinates of the VOIs were allowed to differ dependent on their local maxima, with movement constrained to within 8 mm of the group-level peak." The authors should better clarify this sentence. Has each time course been extracted at the subject level (i.e. time course of subject 1 has been deduced from the ROIs based on the subject 1 GLM’s results) or from group level GLM analysis? I think this information is implicit in the next session but it is not clear. If the time course was extracted at the subject level, the authors should include an image of probabilistic displacement of these ROIs.

Response

We thank the reviewers for highlighting the lack of clarity in this section. We have restructured this sentence to be more explicit about our methodology (page 10, paragraph 2) and included a figure depicting the coordinates of the VOIs.

"For each participant, the center coordinates of these VOIs were dependent on their subject specific local maxima of these regions; these were required to be within 8 mm from the group-level peak (for the resulting distribution of individual coordinates see Figure 1)."

Minor Comments:

1. An image that synthesizes the results in a more user-friendly and intuitive way could

make it easier to read.

Response

We have added color and removed the non-significant associations from Figure 5 to make it easier to comprehend. Thank you for the suggestion.

2. Please better specify the "matching" phase in the experimental design. Once the face has been shown to the top half of the screen, the two faces presented at the bottom half are they sad/fearful expressions or sad or fearful expression plus neutral face?

Response

We have included the following sentence to more explicitly detail the matching phase (page 6, paragraph 2):

"All three faces within a trial (one in the top half and two in the bottom half) displayed the same facial expression."

3. Please indicate the volume of the cluster size that has been reported in the Table (1). This helps the reader to understand the correct size of a cluster.

Response

The volume of the voxels has been added to the cluster size header for Table 1 and 3:

4. Figure 1: please specify the left right orientation of the image (should be right-right orientation)

Response

This figure has been updated with this recommendation (see below).

5. Table 2: please add the significant score in the table and not only in the Behavioural results.

Response

Table 2 has been reconfigured to indicate which behavioral measures were significantly different from one another (see below). The age and gender demographics have been integrated into the text.

"Thus, 92 participants (56.5% female) with a mean age of 20.1 years (S.D. 2.9 years) were included in our analyses."

References

Banks SJ, Eddy KT, Angstadt M, Nathan PJ, Phan KL (2007) Amygdala-frontal connectivity during emotion regulation. Soc Cogn Affect Neurosci 2:303-312.

Barrett LF (2017) The theory of constructed emotion: an active inference account of interoception and categorization. Soc Cogn Affect Neurosci 12:1-23.

Blackford JU, Buckholtz JW, Avery SN, Zald DH (2010) A unique role for the human amygdala in novelty detection. Neuroimage 50:1188-1193.

Braunstein LM, Gross JJ, Ochsner KN (2017) Explicit and implicit emotion regulation: a multi-level framework. Soc Cogn Affect Neurosci 12:1545-1557.

Comte M, Schon D, Coull JT, Reynaud E, Khalfa S, Belzeaux R, Ibrahim EC, Guedj E, Blin O, Weinberger DR, Fakra E (2016) Dissociating Bottom-Up and Top-Down Mechanisms in the Cortico-Limbic System during Emotion Processing. Cereb Cortex 26:144-155.

Corbetta M, Patel G, Shulman GL (2008) The reorienting system of the human brain: from environment to theory of mind. Neuron 58:306-324.

Dannlowski U, Ohrmann P, Konrad C, Domschke K, Bauer J, Kugel H, Hohoff C, Schoning S, Kersting A, Baune BT, Mortensen LS, Arolt V, Zwitserlood P, Deckert J, Heindel W, Suslow T (2009) Reduced amygdala-prefrontal coupling in major depression: association with MAOA genotype and illness severity. Int J Neuropsychoph 12:11-22.

Davey CG, Pujol J, Harrison BJ (2016) Mapping the self in the brain’s default mode network. Neuroimage 132:390-397.

den Ouden H (2012) DCM course in Paris. In: SPM Mailing List.

Dima D, Stephan KE, Roiser JP, Friston KJ, Frangou S (2011) Effective connectivity during processing of facial affect: evidence for multiple parallel pathways. J Neurosci 31:14378-14385.

Dolcos F, Kragel P, Wang LH, McCarthy G (2006) Role of the inferior frontal cortex in coping with distracting emotions. Neuroreport 17:1591-1594.

Etkin A, Buchel C, Gross JJ (2015) The neural bases of emotion regulation. Nature Reviews Neuroscience 16:693.

Fairhall SL, Ishai A (2007) Effective connectivity within the distributed cortical network for face perception. Cereb Cortex 17:2400-2406.

Fusar-Poli P, Placentino A, Carletti F, Landi P, Allen P, Surguladze S, Benedetti F, Abbamonte M, Gasparotti R, Barale F, Perez J, McGuire P, Politi P (2009) Functional atlas of emotional faces processing: a voxel-based meta-analysis of 105 functional magnetic resonance imaging studies. J Psychiatry Neurosci 34:418-432.

Haller SP, Kircanski K, Stoddard J, White LK, Chen G, Sharif-Askary B, Zhang S, Towbin KE, Pine DS, Leibenluft E, Brotman MA (2018) Reliability of neural activation and connectivity during implicit face emotion processing in youth. Dev Cogn Neurosci 31:67-73.

Hariri AR, Tessitore A, Mattay VS, Fera F, Weinberger DR (2002) The amygdala response to emotional stimuli: a comparison of faces and scenes. Neuroimage 17:317-323.

Harrison BJ, Pujol J, Lopez-Sola M, Hernandez-Ribas R, Deus J, Ortiz H, Soriano-Mas C, Yucel M, Pantelis C, Cardoner N (2008) Consistency and functional specialization in the default mode brain network. Proc Natl Acad Sci U S A 105:9781-9786.

Harrison BJ, Pujol J, Contreras-Rodriguez O, Soriano-Mas C, Lopez-Sola M, Deus J, Ortiz H, Blanco-Hinojo L, Alonso P, Hernandez-Ribas R, Cardoner N, Menchon JM (2011) Task-induced deactivation from rest extends beyond the default mode brain network. PLoS One 6:e22964.

Hartley CA, Phelps EA (2010) Changing fear: the neurocircuitry of emotion regulation. Neuropsychopharmacology 35:136-146.

Heberlein AS, Padon AA, Gillihan SJ, Farah MJ, Fellows LK (2008) Ventromedial frontal lobe plays a critical role in facial emotion recognition. J Cogn Neurosci 20:721-733.

Hedger N, Adams WJ, Garner M (2015) Fearful Faces Have a Sensory Advantage in the Competition for Awareness. J Exp Psychol Human 41:1748-1757.

Hiser J, Koenigs M (2018) The Multifaceted Role of the Ventromedial Prefrontal Cortex in Emotion, Decision Making, Social Cognition, and Psychopathology. Biol Psychiatry 83:638-647.

Jacob H, Bruck C, Domin M, Lotze M, Wildgruber D (2014) I can’t keep your face and voice out of my head: neural correlates of an attentional bias toward nonverbal emotional cues. Cereb Cortex 24:1460-1473.

Kiebel SJ, Kloppel S, Weiskopf N, Friston KJ (2007) Dynamic causal modeling: a generative model of slice timing in fMRI. Neuroimage 34:1487-1496.

Kuniecki M, Woloszyn K, Domagalik A, Pilarczyk J (2018) Disentangling brain activity related to the processing of emotional visual information and emotional arousal. Brain Struct Funct 223:1589-1597.

Lin H, Mueller-Bardorff M, Mothes-Lasch M, Buff C, Brinkmann L, Miltner WH, Straube T (2016) Effects of Intensity of Facial Expressions on Amygdalar Activation Independently of Valence. Front Hum Neurosci 10:646.

Markowitsch HJ (1998) Differential contribution of right and left amygdala to affective information processing. Behav Neurol 11:233-244.

Milad MR, Quirk GJ (2012) Fear Extinction as a Model for Translational Neuroscience: Ten Years of Progress. Annu Rev Psychol 63:129-151.

Morris JS, Ohman A, Dolan RJ (1998) Conscious and unconscious emotional learning in the human amygdala. Nature 393:467-470.

Musgrove DR, Eberly LE, Klimes-Dougan B, Basgoze Z, Thomas KM, Mueller BA, Houri A, Lim KO, Cullen KR (2015) Impaired Bottom-Up Effective Connectivity Between Amygdala and Subgenual Anterior Cingulate Cortex in Unmedicated Adolescents with Major Depression: Results from a Dynamic Causal Modeling Analysis. Brain Connectivity 5:608-619.

Palermo R, Rhodes G (2007) Are you always on my mind? A review of how face perception and attention interact. Neuropsychologia 45:75-92.

Raichle ME, MacLeod AM, Snyder AZ, Powers WJ, Gusnard DA, Shulman GL (2001) A default mode of brain function. Proc Natl Acad Sci U S A 98:676-682.

Roy M, Shohamy D, Wager TD (2012) Ventromedial prefrontal-subcortical systems and the generation of affective meaning. Trends Cogn Sci 16:147-156.

Satpute AB, Lindquist KA (2019) The Default Mode Network’s Role in Discrete Emotion. Trends Cogn Sci 23:851-864.

Skerry AE, Saxe R (2015) Neural representations of emotion are organized around abstract event features. Curr Biol 25:1945-1954.

Sladky R, Hoflich A, Kublbock M, Kraus C, Baldinger P, Moser E, Lanzenberger R, Windischberger C (2015) Disrupted Effective Connectivity Between the Amygdala and Orbitofrontal Cortex in Social Anxiety Disorder During Emotion Discrimination Revealed by Dynamic Causal Modeling for fMRI. Cerebral Cortex 25:895-903.

Sreenivas S, Boehm SG, Linden DE (2012) Emotional faces and the default mode network. Neurosci Lett 506:229-234.

Stephan KE, Penny WD, Moran RJ, den Ouden HE, Daunizeau J, Friston KJ (2010) Ten simple rules for dynamic causal modeling. Neuroimage 49:3099-3109.

Todorov A (2012) The role of the amygdala in face perception and evaluation. Motiv Emot 36:16-26.

Vai B, Sferrazza Papa G, Poletti S, Radaelli D, Donnici E, Bollettini I, Falini A, Cavallaro R, Smeraldi E, Benedetti F (2015) Abnormal cortico-limbic connectivity during emotional processing correlates with symptom severity in schizophrenia. Eur Psychiatry 30:590-597.

Viviani R (2014) Neural correlates of emotion regulation in the ventral prefrontal cortex and the encoding of subjective value and economic utility. Front Psychiatry 5:123.

Willinger D, Karipidis, II, Beltrani S, Di Pietro SV, Sladky R, Walitza S, Stampfli P, Brem S (2019) Valence-Dependent Coupling of Prefrontal-Amygdala Effective Connectivity during Facial Affect Processing. eNeuro 6.

Winecoff A, Clithero JA, Carter RM, Bergman SR, Wang L, Huettel SA (2013) Ventromedial prefrontal cortex encodes emotional value. J Neurosci 33:11032-11039.

Winker C, Rehbein MA, Sabatinelli D, Dohn M, Maitzen J, Roesmann K, Wolters CH, Arolt V, Junghoefer M (2019) Noninvasive Stimulation of the Ventromedial Prefrontal Cortex Indicates Valence Ambiguity in Sad Compared to Happy and Fearful Face Processing. Front Behav Neurosci 13:83.

Xia M, Wang J, He Y (2013) BrainNet Viewer: a network visualization tool for human brain connectomics. PLoS One 8:e68910.

Yang M, Tsai SJ, Li CSR (2019) Concurrent amygdalar and ventromedial prefrontal cortical responses during emotion processing: a meta-analysis of the effects of valence of emotion and passive exposure versus active regulation. Brain Structure & Function.

Zhang S, Hu S, Chao HH, Ide JS, Luo X, Farr OM, Li CS (2014) Ventromedial prefrontal cortex and the regulation of physiological arousal. Soc Cogn Affect Neurosci 9:900-908.
